# Explainable machine learning reveals ribosome biogenesis biomarkers in preeclampsia risk prediction

**DOI:** 10.3389/fimmu.2025.1595222

**Published:** 2025-06-09

**Authors:** Jingjing Chen, Dan Zhang, Chengxiu Zhu, Lin Lin, Kejun Ye, Ying Hua, Mengjia Peng

**Affiliations:** ^1^ Department of Gynecology and Obstetrics, The Third Affiliated Hospital of Wenzhou Medical University, Rui’an, China; ^2^ Department of Gynecology and Obstetrics, The Second Affiliated Hospital of Wenzhou Medical University, Wenzhou, China

**Keywords:** preeclampsia, ribosome biogenesis dysregulation, multi-algorithm machine learning, risk model, biomarker validation

## Abstract

**Background:**

Preeclampsia, a hypertensive disorder during pregnancy affecting 2-8% of pregnancies globally, remains a leading cause of maternal and fetal morbidity. Current diagnostic reliance on late-onset clinical features and suboptimal biomarkers underscores the need for early molecular predictors. Ribosome biogenesis, critical for cellular homeostasis, is hypothesized to drive placental dysfunction in PE, though its role remains underexplored.

**Methods:**

We integrated placental transcriptomic data from two datasets (GSE75010, GSE10588) to systematically investigate ribosome biogenesis dysregulation in preeclampsia. Functional enrichment analyses delineated the dysregulation of pathways, while weighted gene co-expression network analysis identified hub genes within ribosome biogenesis-associated modules. A multi-algorithm machine learning framework was employed to optimize predictive performance, with model interpretability achieved through SHapley Additive exPlanations and diagnostic accuracy validated by receiver operating characteristic curves. Immune microenvironment profiling and regulatory network analyses elucidated mechanistic links. Finally, qRT-PCR confirmed the differential expression of key genes in clinical samples.

**Results:**

We identified 25 ribosome biogenesis-related differentially expressed genes, which were significantly enriched in RNA degradation and rRNA processing. Weighted gene co-expression network analysis prioritized seven hub genes. A random forest model incorporating six key feature genes (*GLUL*, *DDX28*, *NCL*, *RIOK1*, *SUV39H1*, *RRS1*) demonstrated robust diagnostic performance, achieving an AUC of 0.972 in the training dataset and 0.917 in the validation dataset. SHapley Additive exPlanations interpretability analysis revealed *SUV39H1* as the dominant risk contributor, while *GLUL* exhibited a protective effect. Regulatory network reconstruction identified 32 transcription factors, 24 RNA-binding proteins, and 62 miRNAs as putative upstream regulators of key genes. Immune Microenvironment Profiling linked key genes to altered placental immune cell populations. qRT-PCR confirmed that *GLUL* and *NCL* expression decreased and *DDX28* and *RIOK1* expression increased in clinical placental samples of preeclampsia group.

**Conclusion:**

This study identifies ribosome biogenesis as one of the pivotal molecular mechanisms to PE pathogenesis, leveraging SHAP-interpretable machine learning to pinpoint six biomarkers. Future research is requisite for the validation of CRISPR and the integration of multi-omics to translate the findings into clinical diagnosis and targeted therapy.

## 1 Introduction

Preeclampsia (PE), a multisystem hypertensive disorder of pregnancy affecting approximately 2-8% of global pregnancies, remains a leading cause of maternal and perinatal morbidity and mortality, accounting for over 70,000 maternal deaths annually with disproportionate impacts in low-resource settings due to limited prenatal care access (1, 2). PE arises from multifactorial interactions between maternal, fetal, and placental components. Central to its pathophysiology is impaired trophoblast function, specifically defective invasion leading to inadequate uterine spiral artery remodeling, which results in shallow placental implantation (3, 4). These placental aberrations induce malperfusion-induced ischemia, endothelial dysfunction, and systemic inflammatory activation, clinically manifesting as gestational hypertension with multiorgan complications. Without timely intervention, progressive disease may culminate in critical maternal complications such as eclampsia and hemolysis, elevated liver enzymes, and low platelet count (HELLP) syndrome (3, 4). Current diagnostic approaches predominantly depend on late-onset clinical features (e.g., proteinuria, hypertension) and suboptimal biomarkers such as soluble fms-like tyrosine kinase-1 to placental growth factor (sFlt-1/PlGF) ratio, which significantly constrains timely clinical intervention. This limitation underscores the critical need for predictive models based on early-stage biomarkers (5, 6).

Ribosome biogenesis is a dynamic, multi-step process involving RNA polymerase I (Pol I)-driven transcription of 47S pre-rRNA, ribosomal protein (RP) assembly, and nucleolar maturation. This fundamental process serves as a pivotal regulator of cellular proteostasis and plays a crucial role in mediating adaptive responses to metabolic and oxidative stress (7). During placental development, ribosome biogenesis is tightly regulated by nutrient-sensing pathways, including MYC-mediated transcriptional activation and mTOR-dependent ribosomal protein synthesis, which collectively coordinate trophoblast proliferation, differentiation, and invasive capacity (7, 44). Dysregulation of ribosome biogenesis disrupts nucleolar architecture, triggering nucleolar stress characterized by impaired rRNA processing, defective ribosomal RNA (rRNA) surveillance, and p53-dependent cell cycle arrest. These molecular perturbations directly contribute to PE’s characteristic pathological features of inadequate placental implantation and vascular dysfunction (8, 44). Moreover, defects in mitochondrial ribosome biogenesis further exacerbate oxidative injury by impairing electron transport chain (ETC) complex assembly, leading to reactive oxygen species (ROS) overproduction and trophoblast apoptosis, as evidenced by downregulated mitochondrial RP expression in PE placentas (8, 45). Concurrently, ribosomopathies reduce translational precision, causing dysregulation of key epithelial-mesenchymal transition (EMT) mediators such as E-cadherin and Snail, thereby suppressing trophoblast migration and spiral artery remodeling (9). These pathological cascades are amplified by epigenetic dysregulation, which represses Pol I activity and exacerbates nucleolar stress (46). The resultant proteostatic imbalance activates compensatory mechanisms such as ribophagy and unfolded protein response (UPR), further depleting functional ribosomes and creating a feedforward loop of placental ischemia and sterile inflammation (47, 48). While these pathophysiological parallels underscore ribosome biogenesis as a critical node in PE pathogenesis, the key feature genes which could predict the PE risk and associated mechanisms remain underexplored, necessitating systematic investigations to translate these insights into biomarkers and treatment targets.

In this study, we hypothesized that dysregulated ribosome biogenesis represents a key molecular driver of PE progression and may serve as a predictive biomarker for PE risk. To address this, we explored the ribosome biogenesis-related differentially expressed genes (RiboRDEGs) in PE and developed a ribosome biogenesis-centric framework for PE risk prediction. Our multi-cohort transcriptomic analysis identified 25 RiboDEGs significantly associated with PE pathogenesis. Functional characterization of these genes revealed their critical roles in PE development. Using weighted gene co-expression network analysis (WGCNA) coupled with ensemble machine learning approaches, we identified six core predictive biomarkers (*GLUL*, *DDX28*, *NCL*, *RIOK1*, *SUV39H1*, and *RRS1*) with high diagnostic potential. SHapley Additive exPlanations (SHAP) analysis elucidated the synergistic contributions of these feature genes to PE risk, while integrated regulatory network analysis uncovered their coordinated transcriptional and post-transcriptional control mechanisms. Importantly, immune microenvironment profiling demonstrated significant associations between key RiboDEGs and altered placental immune cell compositions. These findings provide novel insights into the role of ribosome biogenesis dysregulation in PE pathogenesis. Furthermore, we present a clinically applicable prediction model that bridges molecular mechanisms with early risk assessment, representing a significant advancement toward personalized obstetric care.

## 2 Materials and methods

### 2.1 Data acquisition and preprocessing

Gene expression profiles of PE were retrieved from the National Center for Biotechnology Information (NCBI) Gene Expression Omnibus (GEO) database (7). Three placental tissue-derived Homo sapiens datasets were analyzed: GSE75010 (GPL6244 platform; 80 PE cases and 77 controls) (8–13), GSE10588 (GPL2986 platform; 17 PE cases and 26 controls) (14), and GSE54618 (GPL10558 platform; 12 PE cases and 12 controls) (15) (**Table 1**). Probe annotation was performed for GSE75010 and GSE10588 using platform-specific annotation files, followed by dataset merging and batch effect correction via the sva R package (v3.52.0) (16). The combined dataset (97 PE cases and 103 controls) underwent normalization using the R package limma (v3.60.4) (17), with principal component analysis (PCA) (18) confirming effective batch effect removal. The GSE54618 dataset served as an independent validation cohort, processed identically with probe annotation and normalization.

**Table 1:** GEO microarray chip information.

Series	Platform	Species	Tissue	PE Samples	Control Samples	Cohort
GSE75010	GPL6244	Homo sapiens	Placenta	80	77	Training
GSE10588	GPL2986	Homo sapiens	Placenta	17	26	Training
GSE54618	GPL10558	Homo sapiens	Placenta	12	12	Validation

GEO, Gene Expression Omnibus; PE, Preeclampsia.

### 2.2 Acquisition of ribosome biogenesis-related genes

RiboRGs were systematically acquired through a dual-source approach. Firstly, the GeneCards database (19) was queried using the keyword “ribosome biogenesis,” retaining protein-coding genes with a relevance score >5, which yielded 59 candidate genes. Secondly, 331 RiboRGs were retrieved from the published literature using the same keyword on the PubMed website (20). After merging these two gene lists and removing duplicates, a final set of 344 nonrepetitive RiboRGs was generated for subsequent analysis (**Supplementary Table S1**).

### 2.3 Identification of RiboRDEGs in PE

Differentially expressed genes (DEGs) between PE and control groups were identified using the R package limma with thresholds of |log2 fold change (log2FC) | > 0.1 and adjusted p-value (P.adj) < 0.05 (Benjamini-Hochberg correction). Upregulated and downregulated DEGs were defined as log2FC > 0.1 and log2FC < -0.1, respectively, with statistical significance (P.adj < 0.05). Volcano plots were generated using R package ggplot2 (v3.5.1) to visualize differential expression patterns. RiboRDEGs were subsequently identified by intersecting the DEG list with the precompiled 344 RiboRGs, with results visualized through a Venn diagram. Heatmaps generated by R package ComplexHeatmap (v2.20.0) (21) employed Z-score-normalized counts, hierarchical clustering (Euclidean distance, complete linkage), and three-dimensional PCA maps generated by R package rgl (v1.3.1) demonstrated clear separation of PE and controls.

### 2.4 Functional enrichment analysis

Gene set enrichment analysis (GSEA) (22) was performed on the combined dataset using the R package clusterProfiler (v4.12.6) (23) with the Kyoto Encyclopedia of Genes and Genomes (KEGG) database (24), employing gene set size thresholds of 10–500 genes and significance criteria of P.adj < 0.05 (Benjamini-Hochberg method) and false discovery rate (FDR) < 0.05. To elucidate the biological functions of RiboRDEGs, Gene Ontology (GO) (25), which encompasses biological process (BP), cellular component (CC), and molecular function (MF), and KEGG pathway analysis were performed using clusterProfiler, with the same statistical thresholds. Pathway interaction networks were reconstructed using the R package CBNplot (v1.4.0) (26), where Bayesian networks were inferred through the bnpathplot function by modeling biological pathways as nodes weighted by pathway activity scores derived from RiboRDEG expression profiles.

### 2.5 Weighted gene co-expression network construction and hub gene identification

WGCNA was implemented using the R package WGCNA (v1.73) (27) to identify hub genes among RiboRDEGs. A scale-free topology model was constructed by selecting an optimal soft-thresholding power (β) to maximize network connectivity while minimizing spurious correlations. The adjacency matrix was transformed into a topological overlap matrix (TOM) to quantify gene co-expression similarity, followed by dynamic tree cutting to define gene modules. Module-trait relationships were assessed by calculating Pearson correlation coefficients between module eigengenes (MEs) and PE status, with the most significantly associated module (*p* < 0.05) selected for downstream analysis. Hub genes were identified as the intersection of RiboRDEGs and genes within the PE-correlated module, visualized via Venn diagrams. Pairwise Spearman correlations among hub genes were computed and displayed using the corrplot R package (v0.94).

### 2.6 Predictive model construction and feature gene identification via integrated machine learning

A comprehensive machine learning framework comprising (28) 113 prediction models was developed using the combined dataset to identify key feature genes associated with PE risk. Twelve distinct algorithms spanning linear models (Stepglm, Lasso, Ridge, Enet), ensemble methods (XGBoost, RandomForest, GBM), Bayesian approaches (NaiveBayes), hybrid dimensionality reduction & regularization (plsRglm) and supervised learning techniques (SVM, glmBoost, LDA) were implemented through their respective R packages (glmnet, xgboost, randomForest, etc.), with algorithm combinations detailed in **Supplementary Table S2**. Model performance was evaluated via 10-fold cross-validation, receiver operating characteristic (ROC) curve analysis, and decision curve analysis (DCA), with diagnostic efficacy quantified by mean area under curve (AUC) values across training and validation cohorts (GSE54618). Models demonstrating AUC >0.9 were prioritized as high-diagnostic-value candidates. Final feature gene selection was guided by consensus across top-performing models, validated through calibration curves and confusion matrices to ensure robustness.

### 2.7 Interpretability analysis of optimal predictive model

Model interpretability was assessed using SHAP (29) to delineate the contribution of key feature genes to PE risk prediction. SHAP values were computed via the R package kernelshap (v0.7.0), with positive/negative values indicating directional effects on risk (increase/decrease). Global feature importance rankings were derived from mean absolute SHAP values using shapviz (v0.9.5), visualized through bar plots (overall importance) and beeswarm plots (feature value-SHAP value distributions). SHAP interaction values quantified pairwise feature interdependencies, visualized via scatterplots, while waterfall plots generated for representative cases provided localized interpretability of model decisions. This comprehensive SHAP-based interpretability framework quantifies the importance of key feature genes in predictive model decisions in PE.

### 2.8 ROC curve analysis and protein-protein interaction network analysis of key genes

Key genes were assessed for differential expression between PE and control groups using Mann-Whitney U tests, with results visualized through violin plots. Diagnostic performance was evaluated via ROC curve analysis using the R package pROC (v1.18.5) (30), calculating AUC values to quantify predictive capacity. PPI networks were reconstructed via the GeneMANIA database (31), integrating key genes with functionally associated partners to infer biological modules relevant to PE pathogenesis.

### 2.9 Regulatory network reconstruction

Transcriptional and post-transcriptional regulatory networks were systematically reconstructed to elucidate molecular interactions involving key genes. Transcription factor (TF)-gene interactions were identified using the ChIPBase database (32), retaining TF-gene pairs with combined upstream/downstream supporting samples ≥8. RNA binding protein (RBP) and microRNA (miRNA) interactors were predicted via the ENCORI database (33), applying evidence-based thresholds of clipExpNum ≥10 for RBPs and ≥7 for miRNAs. All interaction networks (TF-gene, RBP-gene, miRNA-gene) were integrated and visualized using Cytoscape (v3.10.2).

### 2.10 Immune microenvironment characterization

Placental immune cell infiltration profiles were quantified via single-sample gene set enrichment analysis (ssGSEA) using the R package GSVA (v1.52.3), with 28 immune cell-specific gene sets derived from established markers (34). Enrichment scores representing relative immune cell abundance were compared between PE and control groups, identifying differentially infiltrated cell types. Spearman correlation matrices generated via the R package linkET (v0.0.7.4) revealed intercellular immune interactions and key gene-immune cell associations (|r| >0.3), visualized through network diagrams. Significant correlations (|r| >0.3) were further validated using scatterplots to delineate linear relationships.

### 2.11 Clinical sample collection and processing

Placental tissue samples were collected from 20 singleton pregnancies (10 PE cases, 10 gestational age-matched controls, each group included five term pregnancy and five preterm pregnancies) undergoing cesarean delivery at the Third Affiliated Hospital of Wenzhou Medical University. PE diagnosis followed ISSHP criteria: sustained hypertension (≥140/90 mmHg) with proteinuria (≥0.3 g/24h) emerging after 20 gestational weeks. Inclusion criteria required maternal age 20–40 years, uncomplicated antenatal course prior to PE onset, and absence of fetal anomalies. Exclusion criteria encompassed pre-existing comorbidities (chronic hypertension, diabetes), acute infections (including COVID-19), gestational diabetes, fetal congenital disorders, and exposure to confounding medications. Full-thickness placental biopsies were obtained from the central region within 15 minutes of delivery, snap-frozen in liquid nitrogen, and stored at −80°C. Ethical approval was granted by the Research Ethics Committee of Ruian People’s Hospital (Approval No. YJ2024178), with written informed consent obtained from all participants.

### 2.12 RNA isolation and quantitative real-time PCR validation

Total RNA was isolated from placental tissues using the Tissue Total RNA Isolation Kit V2 (Vazyme Biotech, RC112-01), with purity and concentration assessed via NanoDrop spectrophotometry (Thermo Fisher Scientific; A260/A280 ratios: 1.8–2.0). Reverse transcription was performed with 1 µg RNA using HiScript III All-in-One RT SuperMix (Vazyme Biotech, R333-01) under optimized conditions: 25°C for 5 min, 50°C for 15 min, and 85°C for 5 min. qRT-PCR assays were conducted on a CFX Connect system (Bio-Rad) with Taq Pro Universal SYBR Master Mix (Vazyme Biotech, Q712-02) in 10 µL reactions (40 cycles: 95°C/10 s denaturation, 60°C/30 s annealing/extension). Melt curve analysis confirmed amplification specificity, and relative gene expression was normalized to GAPDH using the 2−ΔΔCt method. All reactions included triplicate technical replicates, with fold-change calculations relative to control samples.

### 2.13 Statistical analysis

All analyses were conducted within the R statistical environment (v4.4.0). Normality assumptions were verified through Shapiro-Wilk testing, with parametric comparisons (Student’s t-test) applied to normally distributed continuous variables and non-parametric alternatives (Mann-Whitney U test) for skewed distributions. Spearman’s rank correlation coefficient (ρ) quantified associations between molecular features. Unless otherwise stated, all reported p-values were two-tailed, with statistical significance defined as *p* < 0.05. Multiple testing correction was implemented via the Benjamini-Hochberg method for high-throughput datasets to control false discovery rate (FDR < 0.05).

## 3 Results

### 3.1 Analytical flow diagram

**Figure 1:**
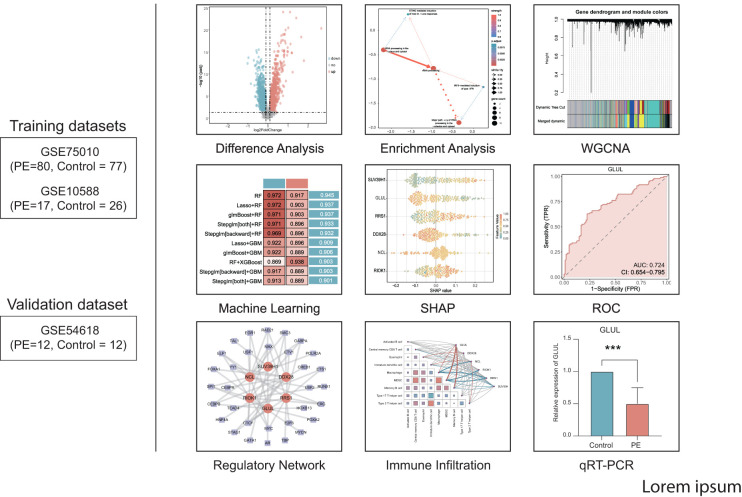
Technology roadmap.

**Figure 1** displays the technical approach of the study, providing a concise overview of the analytical processes used in this study. The analytical flow commenced with merging transcriptomic datasets GSE75010 and GSE10588, followed by identification of DEGs. RiboRGs were intersected with DEGs to derive RiboRDEGs. Subsequent multi-modal enrichment analyses included GSEA, GO, KEGG pathway mapping, and Bayesian network inference to elucidate functional associations. WGCNA identified PE-correlated modules and key genes, while machine learning algorithms refined core diagnostic biomarkers. Model interpretability was enhanced through SHAP, with ROC curve analysis validating diagnostic efficacy using external dataset GSE10588. Immune infiltration profiling via ssGSEA revealed microenvironmental interactions of key genes. Regulatory networks encompassing mRNA-TF, mRNA-miRNA, and mRNA-RBP interactions were reconstructed to delineate molecular mechanisms. Final clinical validation confirmed differential expression patterns of candidate genes in PE cohorts.

### 3.2 Identification of ribosome biogenesis-related differentially expressed genes

**Figure 2:**
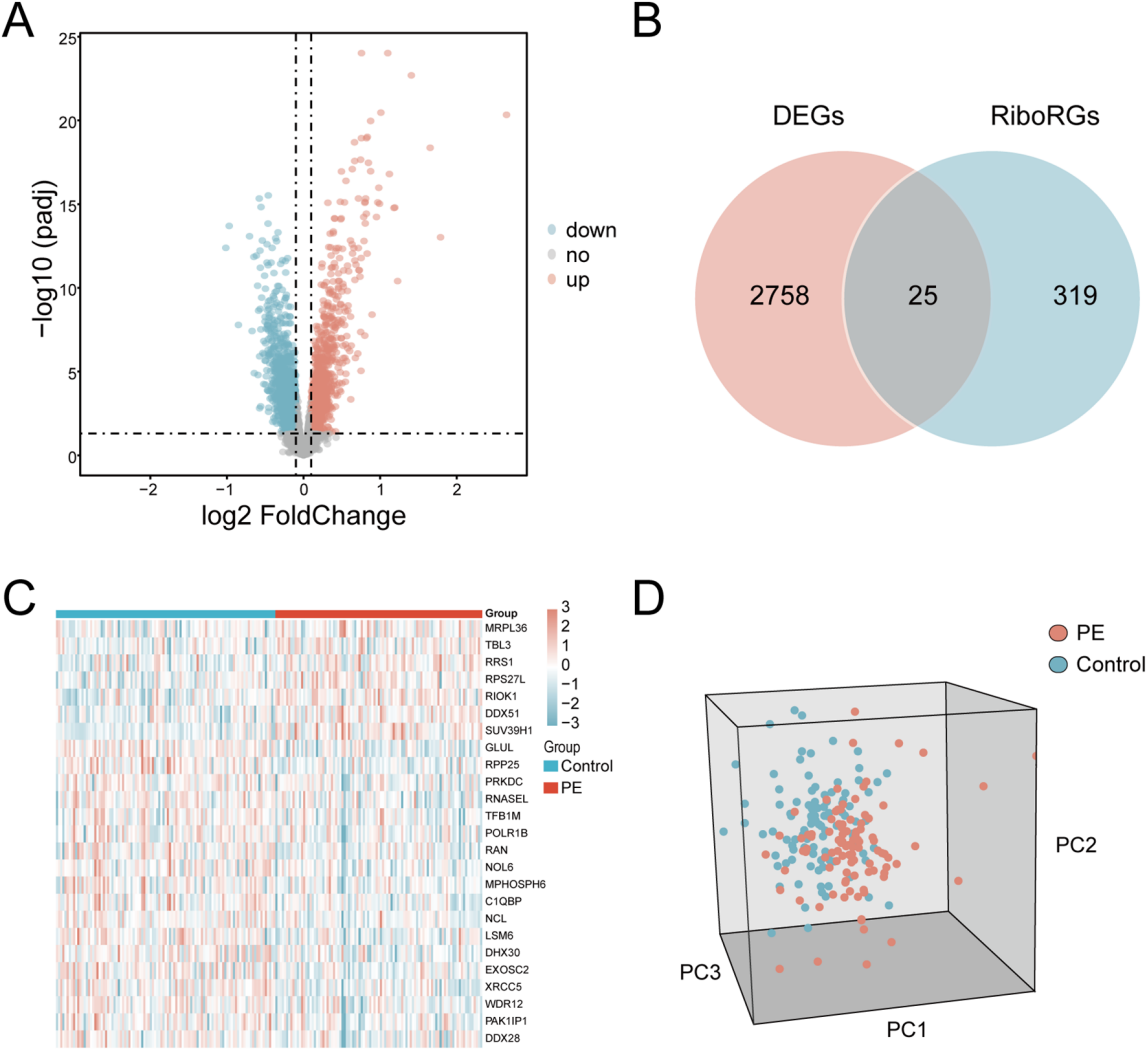
Identification of RiboRDEGs. **(A)** Volcano plot of DEGs (|log2FC| > 0.1, P.adj < 0.05), red indicates upregulated genes and blue indicates downregulated genes **(B)** Venn diagram intersecting DEGs and RiboRGs,red indicates DEGs and blue indicates RiboRGs. **(C)** Heatmap of RiboRDEG expression across samples, red indicates high expression and blue indicates low expression. **(D)** 3D PCA plot demonstrating group segregation based on RiboRDEG profiles, red indicates PE samples, blue indicates normal samples.

The GSE75010 and GSE10588 datasets were merged into a combined cohort (n=200) and subjected to batch effect correction using the R package sva, followed by normalization via the R package limma. Pre-correction boxplots (**Supplementary Figure S1A**) revealed pronounced inter-batch variability in expression distributions, which resolved post-correction (**Supplementary Figure S1B**). PCA demonstrated distinct separation between original datasets along PC1 and PC2 prior to adjustment (**Supplementary Figure S1C**), whereas post-correction PCA (**Supplementary Figure S1D**) showed overlapping clusters with reduced variance contributions (PC1: 2.97%, PC2: 2.19%), confirming effective batch effect mitigation. Based on this combined cohort, we conducted differential expression analysis using the R package limma and identified 2,783 DEGs (|log2FC| > 0.1, P.adj < 0.05) between PE and control groups, comprising 1,304 upregulated and 1,479 downregulated genes (**Figure 2A**). Intersection of these DEGs with the precompiled ribosome biogenesis-related gene set (344 RiboRGs) yielded 25 RiboRDEGs, including *C1QBP*, *DDX28*, *DDX51*, *DHX30*, *EXOSC2*, *GLUL*, *LSM6*, *MPHOSPH6*, *MRPL36*, *NCL*, *NOL6*, *PAK1IP1*, *POLR1B*, *PRKDC*, *RAN*, *RIOK1*, *RNASEL*, *RPP25*, *RPS27L*, *RRS1*, *SUV39H1*, *TBL3*, *TFB1M*, *WDR12* and *XRCC5* (**Figure 2B**). The heatmap reveals the stratified clustering and expression disparity of 25 RiboRDEGs between the PE group and the control group (**Figure 2C**), which is corroborated by 3D PCA demonstrating significant intergroup separation (**Figure 2D**).

### 3.3 Functional enrichment profiling

**Figure 3:**
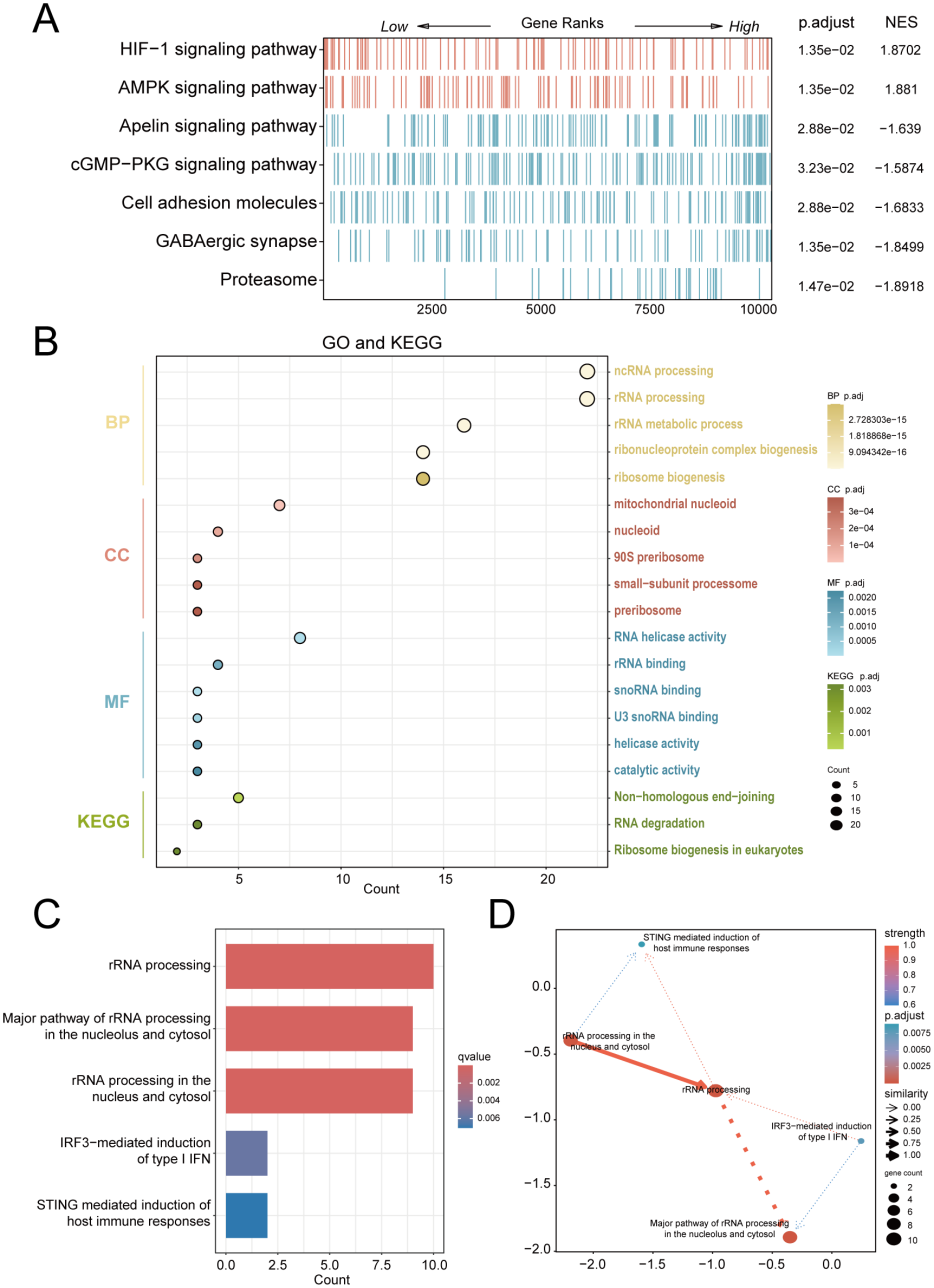
Multi-modal enrichment analysis of PE-associated molecular pathways. **(A)** GSEA heatmap of combined dataset pathways (red: leading-edge enrichment; blue: trailing-edge enrichment). **(B)** Bubble plot of RiboRDEG-enriched GO terms (BP, CC, MF) and KEGG pathways, with term counts on the x-axis. **(C)** Bayesian pathway enrichment bar plot. **(D)** Bayesian network illustrating pathway interactions, with node size reflecting functional centrality and edge properties indicating interaction strength (thickness) and directionality (arrows). Solid/dashed lines distinguish established vs. hypothesized regulatory relationships.

For exploring significantly dysregulated pathways in PE, we conducted GSEA analysis on the combined dataset. Results showed that top enriched terms in PE including HIF-1 signaling, AMPK signaling, and proteasome activity (**Figure 3A**). Subsequent GO and KEGG analyses of the 25 RiboRDEGs demonstrated their predominant involvement in rRNA metabolic processes (BP: ncRNA processing, ribosome biogenesis), nucleolar complexes (CC: 90S preribosome, small-subunit processome), and RNA helicase activities (MF: U3 snoRNA binding) (**Figure 3B**). KEGG pathway enrichment further implicated these genes in ribosome biogenesis and RNA degradation. Bayesian network analysis identified rRNA processing as a central hub (**Figures 3C, D**), exhibiting robust connectivity with immune response pathways (STING-mediated host immunity, IRF3-dependent IFN induction) and subcellular rRNA maturation processes.

### 3.4 Weighted gene co-expression network analysis and hub gene prioritization

**Figure 4:**
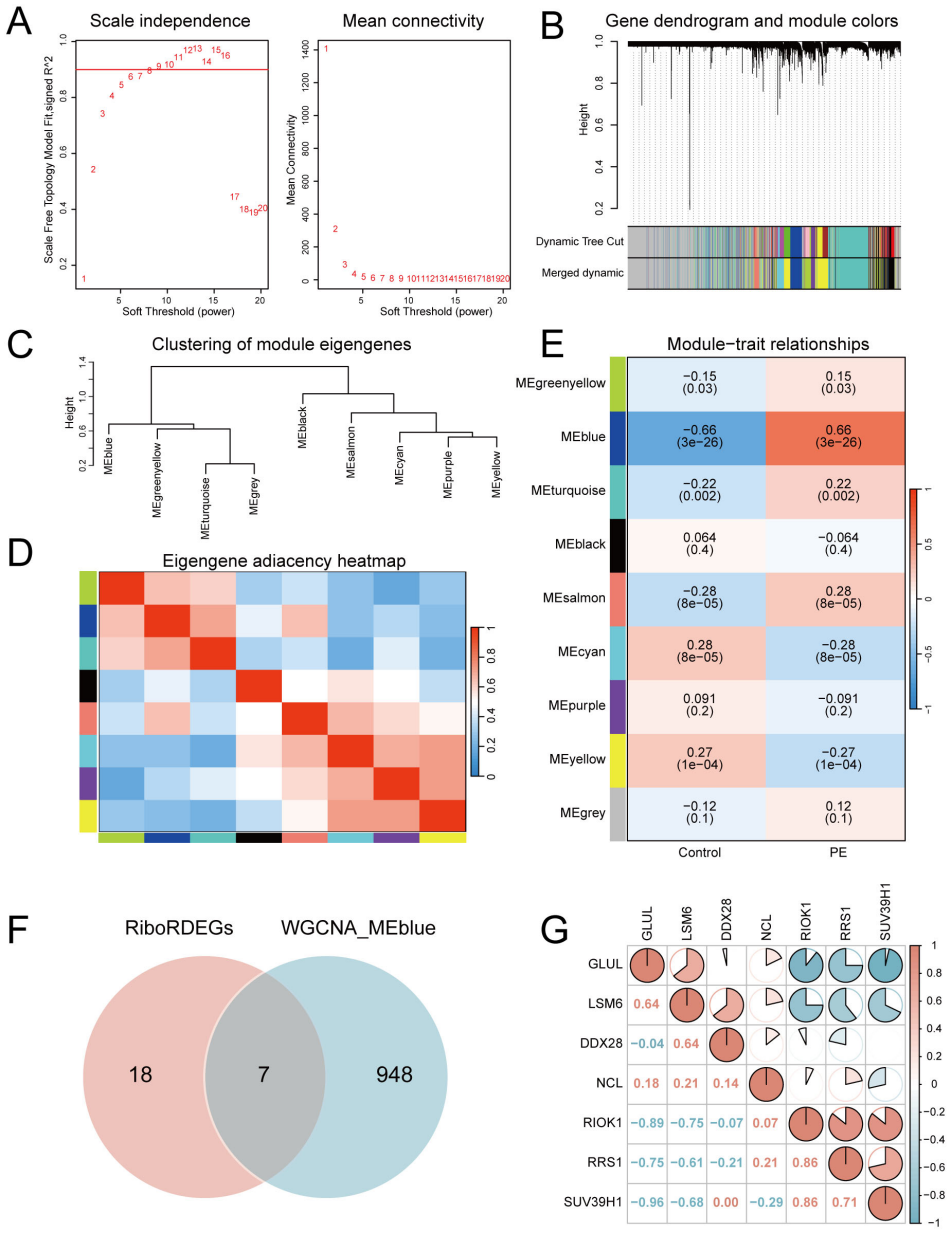
Co-expression network construction and hub gene identification. **(A)** Soft-threshold selection for scale-free topology (left: fit index; right: mean connectivity). **(B)** Hierarchical clustering dendrogram with merged modules. **(C)** Module eigengene clustering tree. **(D)** Inter-module correlation heatmap. **(E)** Module-trait correlation heatmap highlighting MEblue-PE association. (upper: correlation coefficients; lower: p-values) **(F)** Venn diagram intersecting MEblue genes and RiboRDEGs. **(G)** Correlation heatmap of seven hub genes. In the heatmap, red indicates a positive correlation and blue indicates a negative correlation. |r|>0.95: significant correlation; |r|≥0.8: highly correlated; 0.5≤|r|<0.8: moderately correlated; 0.3≤|r|<0.5: weakly correlated; |r|<0.3: not correlated.

A scale-free co-expression network was constructed using WGCNA (power = 10, scale-free fit R² = 0.9; **Figure 4A**). Dynamic module detection identified 9 gene clusters (**Figures 4B, C**), with low inter-module correlations confirmed by a heatmap analysis (**Figure 4D**). The MEblue module exhibited the strongest positive correlation with PE (cor = 0.66, *p* = 3×10^−26^; **Figure 4E**). Intersection of MEblue module genes (n=955) with RiboRDEGs revealed seven hub genes (*GLUL*, *LSM6*, *DDX28*, *NCL*, *RIOK1*, *RRS1*, *SUV39H1*; **Figure 4F**). Spearman correlation analysis demonstrated strong co-expression patterns among hub genes (**Figure 4G**), suggesting functional synergy in PE pathogenesis.

### 3.5 Machine learning-based predictive model construction and validation

**Figure 5:**
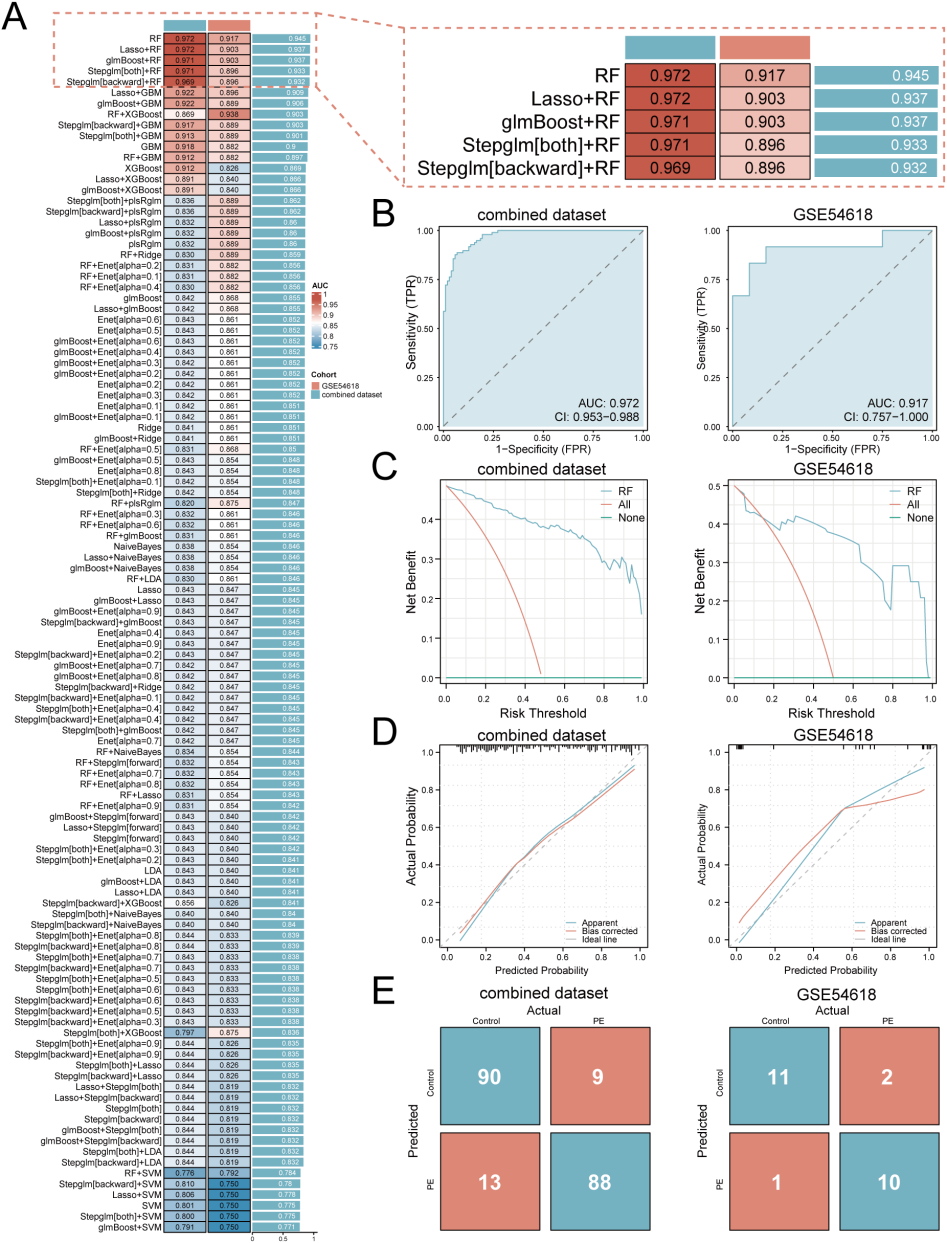
Development and validation of the machine learning-based predictive model. **(A)** Bar plot comparing mean AUCs of 113 algorithm combinations across training and validation cohorts. **(B)** ROC curves of the RF model (TPR, true positive rate; FPR, false positive rate; AUC>0.9 indicates high diagnostic value). **(C)** Decision curve analysis evaluating clinical utility. **(D)** Calibration curves assessing prediction accuracy. **(E)** Confusion matrices quantifying classification performance.

A multi-algorithm framework comprising 113 model combinations was implemented to identify optimal predictors of PE risk using seven hub genes (**Figure 5A**). Random Forest (RF) demonstrated superior diagnostic performance, achieving AUCs of 0.972 (95% CI: 0.953–0.988) in the training cohort and 0.917 (95% CI: 0.757–1.000) in the validation cohort (**Figure 5B**). The final RF model incorporated six feature genes (*GLUL*, *DDX28*, *NCL*, *RIOK1*, *SUV39H1*, *RRS1*; **Table 2**). DCA shows that this model can provide substantial clinical net benefits for clinical decision making (**Figure 5C**), while calibration curves showed strong concordance between predicted and observed PE probabilities (**Figure 5D**). Confusion matrices further validated the model’s diagnostic accuracy, achieving sensitivity >90% and specificity >85% in both training and validation cohorts (**Figure 5E**).

**Table 2:** Description of RiboRDEGs.

ID	Description	log2FC	AveExpr	t	adj.P.Val	B
*GLUL*	*Glutamate-Ammonia Ligase*	-0.37296	11.51056	-5.9997	3.05E-07	9.640216
*DDX28*	*DEAD-Box Helicase 28*	-0.10067	8.489916	-2.97947	0.014957	-2.47268
*NCL*	*Nucleolin*	-0.13118	11.62773	-3.11307	0.010559	-2.08675
*RIOK1*	*RIO Kinase 1*	0.173268	9.869928	5.106692	1.42E-05	5.379976
*SUV39H1*	*SUV39H1 Histone Lysine Methyltransferase*	0.293488	8.689993	6.640665	1.39E-08	12.98921
*RRS1*	*Ribosome Biogenesis Regulator 1 Homolog*	0.144664	9.323376	3.859158	0.001257	0.344918

RiboRDEGs, ribosome biogenesis-related differentially expressed genes.

**Figure 6:**
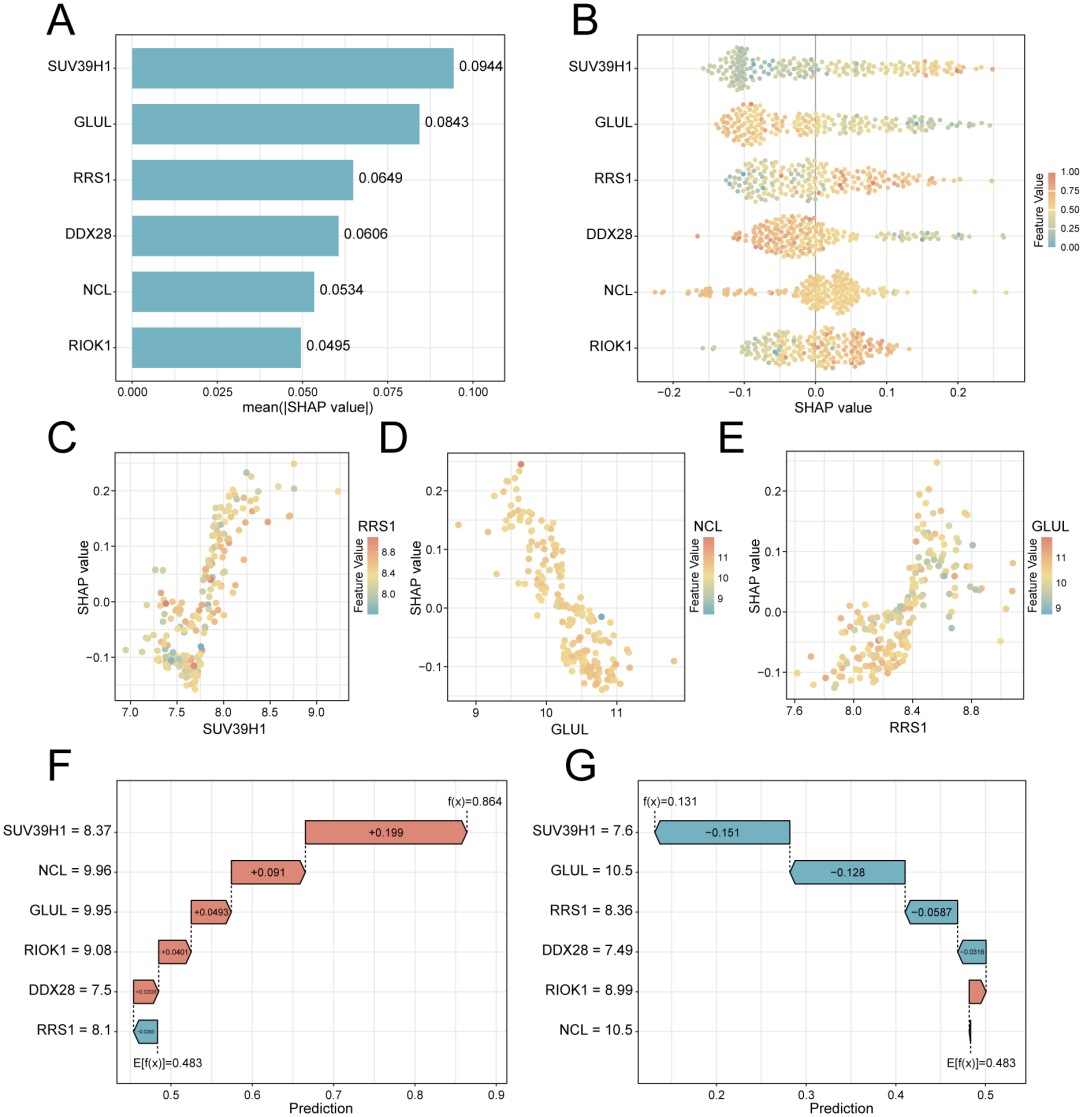
SHAP-based interpretation of key feature genes in the RF predictive model. **(A)** Feature importance bar plot ranked by mean absolute SHAP values. **(B)** Beeswarm plot showing SHAP value directionality (x-axis) versus gene expression (color scale: red = high, blue = low). **(C–E)**. Interaction scatterplots: x-axis = gene expression, y-axis = SHAP value, color = interacting gene expression. **(F)** Waterfall plot for a PE case (predicted risk = 0.864). **(G)** Control case waterfall plot (risk = 0.131). Arrows indicate SHAP contributions (red: risk increase; blue: decrease).

To elucidate the potential impact of the six feature genes on the risks associated with PE, we conducted SHAP analysis. This analysis revealed *SUV39H1* as the most influential feature in the RF model (mean |SHAP| = 0.0944), followed by *GLUL* (|SHAP| = 0.0843), *RRS1* (mean |SHAP| = 0.0649), *DDX28* (mean |SHAP| = 0.0606), *NCL* (mean |SHAP| = 0.0534) and *RIOK1* (mean |SHAP| = 0.0495) (**Figure 6A**). The expression levels of these genes have different directional effects on PE risk. Elevated *SUV39H1*, *RRS1* and *RIOK1* expression correlated with increased PE probability, whereas higher *GLUL*, *DDX28* and *NCL* levels exhibited protective effects (**Figure 6B**). Interaction analysis identified synergistic risk amplification between *SUV39H1* and *RRS1* (**Figure 6C**), while *GLUL*-*NCL* co-expression showed concerted risk reduction (**Figure 6D**). A negative interaction between *RRS1* and *NCL* (**Figure 6E**) suggested compensatory regulatory dynamics. Waterfall plots for representative PE (predicted probability = 0.864) and control (probability = 0.131) cases demonstrated model interpretability, with *SUV39H1* contributing most substantially to risk prediction (**Figures 6F, G**).

### 3.6 ROC curve analysis and protein interaction network of key genes

**Figure 7:**
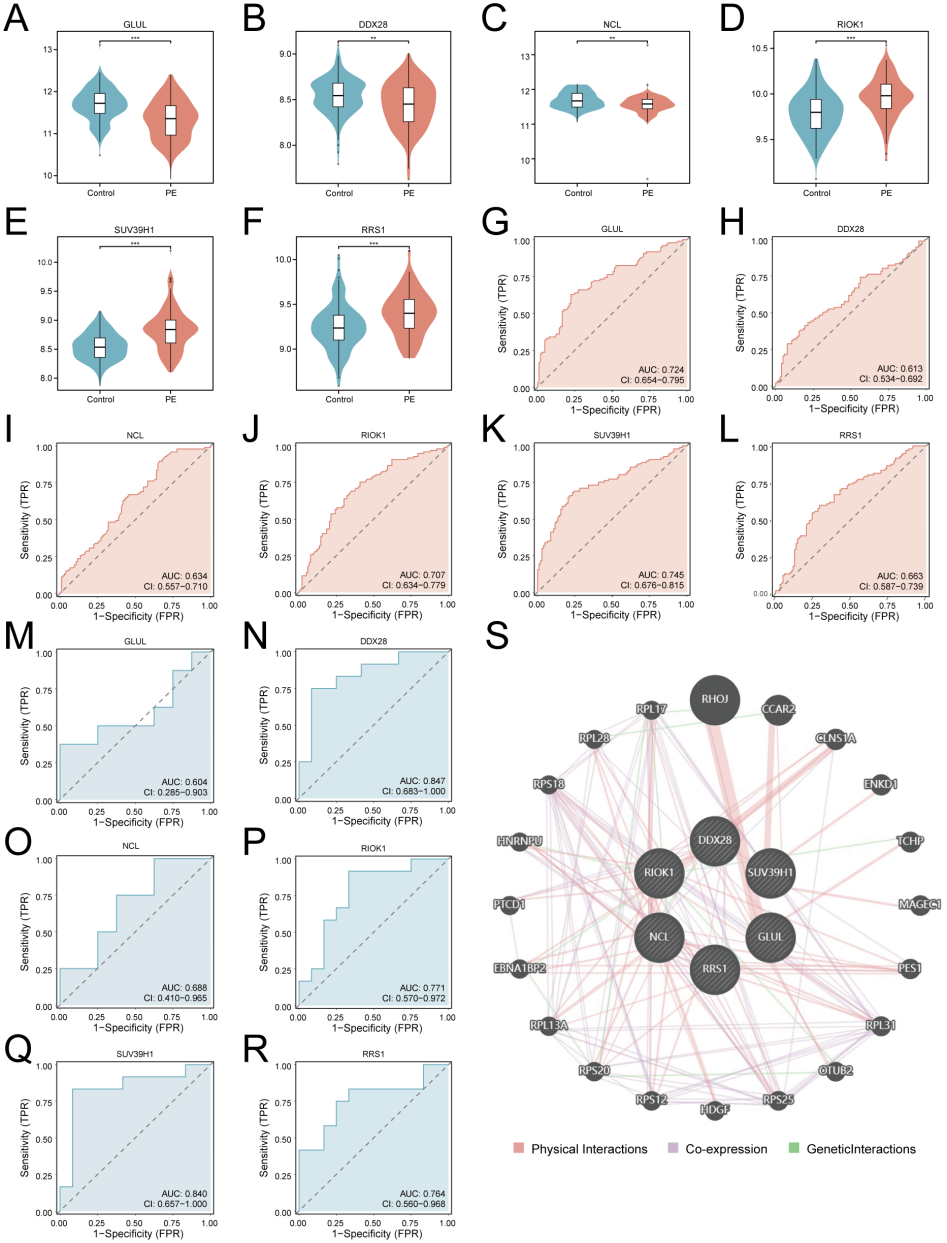
Diagnostic validation and functional interaction networks of key genes. **(A–F)**. Violin plots of key gene expression in PE (red) vs. controls (blue): *GLUL* **(A)**, *DDX28* **(B)**, *NCL* **(C)**, *RIOK1* **(D)**, *SUV39H1* **(E)**, *RRS1* **(F)**. G-L. ROC curves for training cohort: *GLUL* **(G)**, *DDX28* **(H)**, *NCL* **(I)**, *RIOK1* **(J)**, *SUV39H1* **(K)**, *RRS1* **(L)**. **(M–R)**. Validation cohort ROC curves: *GLUL* **(M)**, *DDX28* **(N)**, *NCL* **(O)**, *RIOK1* **(P)**, *SUV39H1* **(Q)**, *RRS1* **(R)**. An AUC of 0.5–0.7 indicates low diagnostic utility, while an AUC of 0.7–0.9 suggests moderate diagnostic value. **(S)** PPI network (nodes: key genes and functionally associated partners; edges: physical interactions (red), co-expression (purple), geneticlnteractions (green); thickness: interaction confidence). ***p < 0.001; **p < 0.01.

Differential expression analysis confirmed significant downregulation of *GLUL*, *DDX28*, and *NCL* (*p* < 0.01; **Figures 7A–C**) alongside upregulation of *RIOK1*, *SUV39H1*, and *RRS1* (*p* < 0.001; **Figures 7D–F**) in PE placentas. ROC analysis revealed moderate diagnostic utility for *GLUL* (AUC = 0.724), *RIOK1* (AUC = 0.707), and *SUV39H1* (AUC = 0.745) in the training cohort, with weaker performance for *DDX28* (AUC = 0.613), *NCL* (AUC = 0.634), and *RRS1* (AUC = 0.663) (**Figures 7G–L**). External validation in GSE54618 maintained moderate diagnostic accuracy for *DDX28* (AUC = 0.847), *RIOK1* (AUC = 0.771), *SUV39H1* (AUC = 0.840), and *RRS1* (AUC = 0.764), while *GLUL* (AUC = 0.604) and *NCL* (AUC = 0.688) showed limited discriminative power (**Figures 7M–R**). Protein-protein interaction (PPI) network analysis via GeneMANIA identified 20 functionally associated partners (**Figure 7S**), with co-expression (47.61%) and physical interactions (51.88%) as predominant interaction modes, suggesting that these key genes may collaboratively regulate ribosome biogenesis and RNA processing through transcriptional coordination and direct molecular binding, thereby contributing to placental dysfunction in PE pathogenesis.

### 3.7 Reconstruction of regulatory networks

To explore the systematic regulatory network of ribosome biogenesis in preeclampsia, the reconstruction of regulatory networks was conducted. Transcriptional and post-transcriptional regulatory networks were systematically mapped to elucidate molecular interactions involving key genes. The TF network, constructed using ChIPBase with a stringent filtering criterion (combined upstream/downstream supporting samples ≥8), comprised 6 key genes (*GLUL*, *DDX28*, *NCL*, *RIOK1*, *SUV39H1*, *RRS1*) interacting with 32 TFs through 59 regulatory pairs (**Supplementary Figure S2A**, **Supplementary Table S3**). RBP interactions predicted via ENCORI (clipExpNum ≥10) revealed 45 functional associations between 6 key genes (*GLUL*, *DDX28*, *NCL*, *RIOK1*, *SUV39H1*, *RRS1*) and 24 RBPs (**Supplementary Figure S2B**, **Supplementary Table S4**). miRNA-mediated regulation analysis identified 63 mRNA-miRNA pairs involving *GLUL*, *SUV39H1*, and *NCL* with 62 miRNAs (clipExpNum ≥7; **Supplementary Figure S2C**, **Supplementary Table S5**), highlighting transcriptional and post-transcriptional modulation of ribosome biogenesis pathways in PE.

### 3.8 Immune microenvironment profiling via ssGSEA

**Figure 8:**
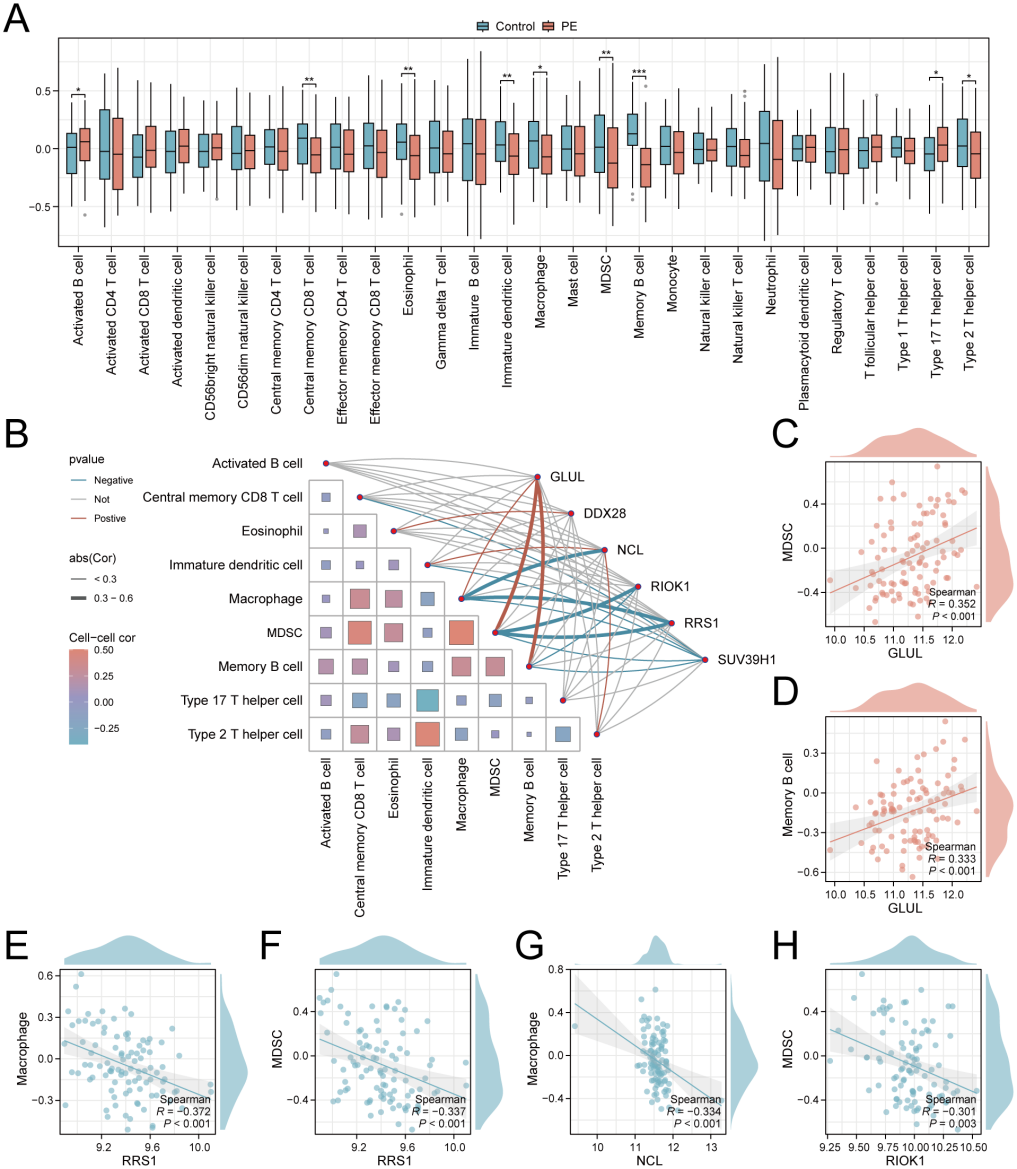
Immune infiltration landscape and key gene correlations in PE. **(A)** Boxplots comparing immune cell enrichment scores between PE (red) and controls (blue). **(B)** Correlation network of immune cells and key genes. **(C–H)**. Scatterplots of key gene-immune cell correlations: *GLUL*-MDSC **(C)**, *GLUL*-Memory B cell **(D)**, *RRS1*-Macrophage **(E)**, *RRS1*-MDSC **(F)**, *NCL*-Macrophage **(G)**, *RIOK1*-MDSC **(H)**. *p < 0.05; **p < 0.01; ***p < 0.001. red indicates a positive correlation and blue indicates a negative correlation. 0.3≤|r|<0.5: weakly correlated.

To explore the role of Ribosome biogenesis in the immune microenvironment of PE, we conducted an ssGSEA analysis on the combined dataset. ssGSEA revealed significant dysregulation of nine immune cell types in PE placentas compared to controls (*p* < 0.05; **Figure 8A**). Activated B cells and Th17 cells were enriched in PE, while central memory CD8+ T cells, eosinophils, immature dendritic cells, macrophages, MDSCs, memory B cells, and Th2 cells exhibited reduced infiltration. Correlation network analysis demonstrated interconnected immune cell dynamics in PE, with key genes showing cell-type-specific associations (**Figure 8B**). Notably, *GLUL* expression positively correlated with MDSC (r = 0.352, *p* < 0.001; **Figure 8C**) and memory B cell abundance (r = 0.333, *p* < 0.001; **Figure 8D**). Conversely, *RRS1* displayed negative correlations with macrophages (r = -0.372, *p* < 0.001; **Figure 8E**) and MDSCs (r = -0.337, *p* < 0.001; **Figure 8F**), while *NCL* and *RIOK1* inversely associated with macrophage (r = -0.334, *p* < 0.001; **Figure 8G**) and MDSC infiltration (r = -0.301, *p* = 0.003; **Figure 8H**), respectively.

### 3.9 Experimental validation of key genes in PE

**Figure 9:**
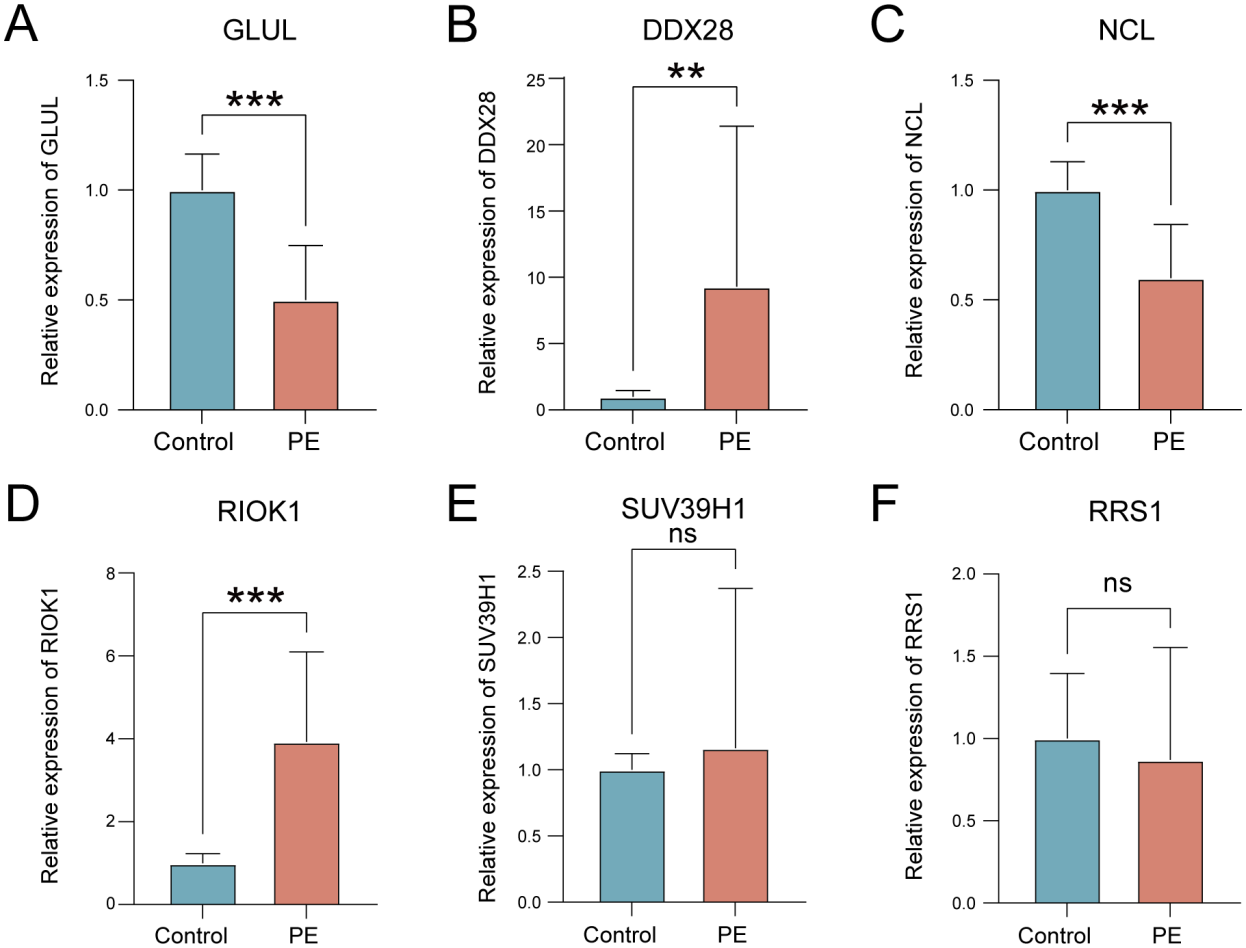
Validation of key gene expression in PE placental tissues. **(A–F)** mRNA expression levels of *GLUL* **(A)**, *DDX28* **(B)**, *NCL* **(C)**, *RIOK1* **(D)**, *SUV39H1* **(E)**, and *RRS1* **(F)** in control (blue) versus PE (red) groups. ns, not significant; **p < 0.01; ***p < 0.001.

qRT-PCR validation in placental tissues from 10 PE patients and 10 gestational age-matched controls (primers listed in **Table 3**) confirmed significant dysregulation of ribosome biogenesis-associated genes. *DDX28* (*p* < 0.01) and *RIOK1* (*p* < 0.001) expression was markedly elevated in PE placentas, while *GLUL* (*p* < 0.001) and *NCL* (*p* < 0.001) showed significant downregulation (**Figures 9A–D**). *SUV39H1* and *RRS1* exhibited non-significant expression trends (**Figures 9E, F**). Clinical assessment revealed significantly elevated systolic blood pressure in PE cases compared to gestational age-matched controls (155.1 ± 24.27 vs. 122.6 ± 7.91 mmHg, *p* < 0.001), with diastolic pressures similarly increased (92.9 ± 9.73 vs. 74.4 ± 3.69 mmHg, *p* < 0.001). No significant differences were observed in maternal age (30.4 ± 5.21 vs. 29.7 ± 4.60 years, *p* = 0.754), neonatal birth weight (2,724 ± 500.16 vs. 3,014 ± 503.42 g, *p* = 0.213), or Apgar scores at 1 min (9: 10% vs. 0%, *p* = 0.305) and 5 min (9: 10% vs. 0%, *p* = 0.305) (**Table 4**).

**Table 3:** Primer sequences for qRT-PCR.

Gene	Primer sequences (5′-3′)
*GLUL*	AAGAGTTGCCTGAGTGGAATTTC (forward) AGCTTGTTAGGGTCCTTACGG (reverse)
*DDX28*	TGCGAAAGCTCTCGTCTAAGG (forward) CCTCCTGTAGTGCGTGCAG (reverse)
*NCL*	GCACCTGGAAAACGAAAGAAGG (forward) GAAAGCCGTAGTCGGTTCTGT (reverse)
*RIOK1*	GGCTCGGGAGTTGTACCTG (forward) CCACGGACTGAGACACGTC (reverse)
*SUV39H1*	CCTGCCCTCGGTATCTCTAAG (forward) ATATCCACGCCATTTCACCAG (reverse)
*RRS1*	GTTACCTCCCGTTTCCCACTT (forward) CATCACCGATTGGTCATCTCTTG (reverse)
*GAPDH*	TGTGGGCATCAATGGATTTGG (forward) ACACCATGTATTCCGGGTCAAT (reverse)

qRT−PCR, quantitative real−time PCR.

**Table 4:** Clinical information of the patients.

Category	PE (n = 10)	Control (n = 10)	*p*-value
Gestational age at delivery (weeks)	36.1 ± 1.66	36.1 ± 1.66	1.000
Age (years)	30.4 ± 5.21	29.7 ± 4.60	0.754
Systolic blood pressure (mmHg)	155.1 ± 24.27	122.6 ± 7.91	< 0.001
Diastolic blood pressure (mmHg)	92.9 ± 9.73	74.4 ± 3.69	< 0.001
Neonatal birth weight (g)	2724 ± 500.16	3014 ± 503.42	0.213
1 min Apgar (score)			0.305
10	9 (90%)	10 (100%)	
9	1 (10%)	0 (0%)	
5 min Apgar (score)			0.305
10	9 (90%)	10 (100%)	
9	1 (10%)	0 (0%)	

## 4 Discussion

Conventional PE biomarkers, including PlGF and sFlt-1, exhibit limited predictive accuracy during early gestation due to insufficient sensitivity and specificity (6). These markers fail to resolve molecular heterogeneity across clinical PE subtypes and primarily reflect angiogenic imbalance while neglecting synergistic pathogenic mechanisms such as inflammatory and metabolic dysregulation (35). Emerging evidence indicates that ribosome biogenesis dysregulation is closely associated with the core PE pathological features, such as placental malperfusion and aberrant vascular remodeling, by disrupting nucleolar structural integrity, inducing oxidative stress, and impairing trophoblast function (36, 37). These evidences collectively suggest that the disruption of ribosome homeostasis may represent a pivotal molecular hub in the early PE pathogenesis.

Our analysis reveals that RiboRDEGs are significantly enriched in rRNA metabolic processes (including ncRNA processing and 90S preribosome assembly) and show strong Bayesian network connectivity to immune pathways (STING/IRF3-mediated interferon responses). These findings suggest that ribosome biogenesis defects in PE may drive placental dysfunction through dual mechanisms: (1) impaired ribosomal stress adaptation via disrupted rRNA maturation (38), and (2) immune activation triggered by nucleolar-derived damage-associated molecular patterns (DAMPs) (39, 40). The co-enrichment of HIF-1 and AMPK signaling pathways aligns with placental hypoxia-reperfusion injury in PE, where hypoxia-inducible factors may suppress rRNA transcription while energy stress activates AMPK-mediated ribophagy to eliminate defective ribosomes (41, 42). Additionally, KEGG pathway analysis demonstrates concurrent proteasome activation, likely representing a compensatory mechanism to remove misfolded ribosomal proteins generated during biogenesis stress, a process previously linked to PE-associated oxidative injury (40, 43). These findings collectively position ribosome biogenesis as a nexus integrating metabolic stress, proteostatic imbalance, and sterile inflammation in PE pathogenesis. The identified RiboDEGs emerge as key molecular mediators linking nucleolar dysfunction to clinical disease manifestations.

Analysis of the combined dataset revealed distinct immune dysregulation in PE placentas, characterized by Th17 polarization, MDSC depletion, and impaired macrophage infiltration, which is consistent with the Th17/Th2 imbalance feature of PE (44). These immunological alterations might represent secondary outcome of ribosome biogenesis stress and the release of DAMPs due to nuclear instability. These DAMPs engage cytoplasmic sensors (e.g., RIG-I, MDA5) and toll-like receptors (TLRs) on placental immune cells, driving NF-κb-dependent pro-inflammatory cytokines (IL-6, IL-17), while suppressing anti-inflammatory mediators (IL-10, TGF-β). This imbalance establishes a self-sustaining inert inflammatory cycle and activates the toll-like receptor (TLR)-mediated inflammatory cascade reaction (39, 44). Notably, *GLUL* expression positively correlated with MDSC and memory B cell abundance, suggesting glutamine synthetase activity may modulate immunosuppressive niches, potentially through mTORC1-dependent metabolic reprogramming of myeloid cells (45). Conversely, *RRS1* and *NCL* showed negative associations with macrophages and MDSCs, implicating their roles in restraining pro-inflammatory polarization, possibly via ER stress pathways that regulate phagocytic clearance of ribosomal debris (40). The coordinated depletion of tolerogenic MDSCs and macrophages further exacerbates vascular dysfunction, creating a feedforward loop between ribosomal stress, oxidative injury, and immune-mediated endothelial damage (46).

To elucidate the predictive potential of RiboRDEGs in PE risk, we pioneered an explainable machine learning (XML) approach that integrates ribosome biogenesis biology with advanced computational modeling to address the critical need for mechanistically interpretable biomarkers in PE risk prediction. By implementing a multi-algorithm framework encompassing 113 model combinations, we developed a RF model that achieves exceptional diagnostic accuracy while maintaining biological interpretability, representing a significant advance over conventional ‘black-box’ approaches (47). The SHAP interpretability analysis revealed *SUV39H1* as the dominant risk contributor (mean |SHAP|=0.0944), with *GLUL* and *RRS1* exhibiting counteractive protective/risk effects, demonstrating how XML disentangles complex gene interactions that collectively drive PE pathogenesis (48). This approach successfully identified six ribosome biogenesis-related biomarkers (*GLUL*, *DDX28*, *NCL*, *RIOK1*, *SUV39H1* and *RRS1*) and mapped their nonlinear synergies, such as the risk-amplifying *SUV39H1*-*RRS1* interaction and protective *GLUL*-*NCL* axis. This findings provide unprecedented insights into how ribosomal stress pathways coalesce to induce placental dysfunction.

The clinical validity of our model was comprehensively demonstrated through two complementary approaches: decision curve analysis confirmed substantial net benefit across clinically relevant risk thresholds, and protein-protein interaction networks revealed these biomarkers functionally coordinate ribosome biogenesis through both physical binding and co-expression relationships. While individual genes showed moderate diagnostic power, their ensemble performance underscores the necessity of multi-gene panels for capturing PE’s molecular heterogeneity — an optimization of current single-biomarker approaches such as the sFlt-1/PlGF ratio (6). Our XML-driven strategy bridges the critical gap between computational prediction and biological mechanism, offering both a clinically deployable risk assessment tool and a systems-level understanding of ribosomal dysregulation in PE pathogenesis (49).

While our explainable machine learning framework provides novel insights into ribosome biogenesis biomarkers for PE risk prediction, several limitations warrant consideration. First, the retrospective design and reliance on public placental transcriptomic datasets may introduce selection bias, as these lack detailed clinical subtyping (e.g., early- vs. late-onset PE) and longitudinal samples to track biomarker dynamics across gestation. Second, the modest clinical validation cohort limits statistical power to detect subtle expression differences, potentially explaining non-significant qRT-PCR trends for *SUV39H1* and *RRS1*. Third, while the model shows promising cross-cohort performance, its generalizability requires testing in multi-ethnic populations and early-pregnancy blood samples, given the inaccessibility of placental biopsies for prenatal screening. Additionally, the exclusive focus on transcriptional regulation overlooks post-translational modifications (e.g., phosphorylation) and epigenetic mechanisms modulating ribosome biogenesis. Furthermore, SHAP-derived gene interactions remain hypothetical without experimental confirmation through CRISPR-based functional validation in trophoblast models. To address these gaps, future studies need to further validate the mechanistic contribution of key biomarkers through functional experiments (such as CRISPR gene editing and ribosome dynamic analysis) and build a multi-omics integration framework — using single-cell transcriptomes to analyze the specific regulatory network of placental trophoblast/immune cell subsets and combining spatial transcriptomes to map the spatial distribution of ribosomal stress signals in the microenvironment. The effect of post-translational modifications (such as phosphorylation) on ribosome assembly was quantitatively analyzed by the proteome, and the synergistic regulatory pattern of DNA methylation/histone modification was characterized by the epigenome. Multi-dimensional data (metabolome, cell free RNA) of prenatal longitudinal blood samples were further integrated to establish a dynamic risk prediction model based on machine learning. This systematic biological strategy from molecular mechanism to clinical phenotype will reveal the cross-scale regulation of PE development driven by the imbalance of ribosome quality control and then promote the transformation process of biomarkers into clinical diagnostic tools.

## 5 Conclusion

This study establishes dysregulation of ribosome biogenesis as one of the pivotal molecular mechanisms underlying the pathogenesis of PE and leveraging XML to identify clinically actionable biomarkers. Through multi-cohort transcriptomic integration, we identified 25 RiboRDEGs, with six hub genes (*GLUL*, *DDX28*, *NCL*, *RIOK1*, *SUV39H1*, *RRS1*) forming the core of a high-performance predictive model (AUC >0.9). SHAP interpretability analysis revealed *SUV39H1* as the dominant risk contributor, while *GLUL* and *NCL* exhibited protective effects, highlighting bidirectional regulatory dynamics in placental stress adaptation. Functional enrichment and Bayesian network analyses linked these genes to rRNA processing, nucleolar stress, and immune dysregulation, with immune microenvironment profiling demonstrating significant correlations between RiboRDEGs and altered placental immune cell populations (e.g., MDSCs, macrophages). Experimental validation confirmed dysregulation of key genes. Despite these advances, our study has limitations including retrospective design, potential selection bias in public datasets, and modest validation cohort size. Future work requires CRISPR-based functional validation of key biomarkers and multi-omics integration (single-cell/spatial transcriptomics, proteomics, epigenomics) to map the mechanism of ribosome biogenesis. Development of blood-based machine learning models incorporating longitudinal metabolomic/cfRNA data could enable dynamic risk prediction. Elucidating post-translational modifications (e.g., phosphorylation) and epigenetic regulation of ribosome biogenesis will clarify cross-scale mechanisms underlying PE pathogenesis. These efforts will bridge ribosome biogenesis insights to clinical translation, advancing early diagnosis and targeted therapies for PE.

## Data Availability

Publicly available datasets were analyzed in this study. This data can be found here: https://www.ncbi.nlm.nih.gov/geo/.
