# Supplementary material for: Explainable machine learning reveals ribosome biogenesis biomarkers in preeclampsia risk prediction
Source: Front Immunol. 2025 Jun 9;16:1595222. doi: 10.3389/fimmu.2025.1595222 (PMC12183210; doi:10.3389/fimmu.2025.1595222)
Supplement: Supplementary file 1 [file DataSheet1.docx]

**
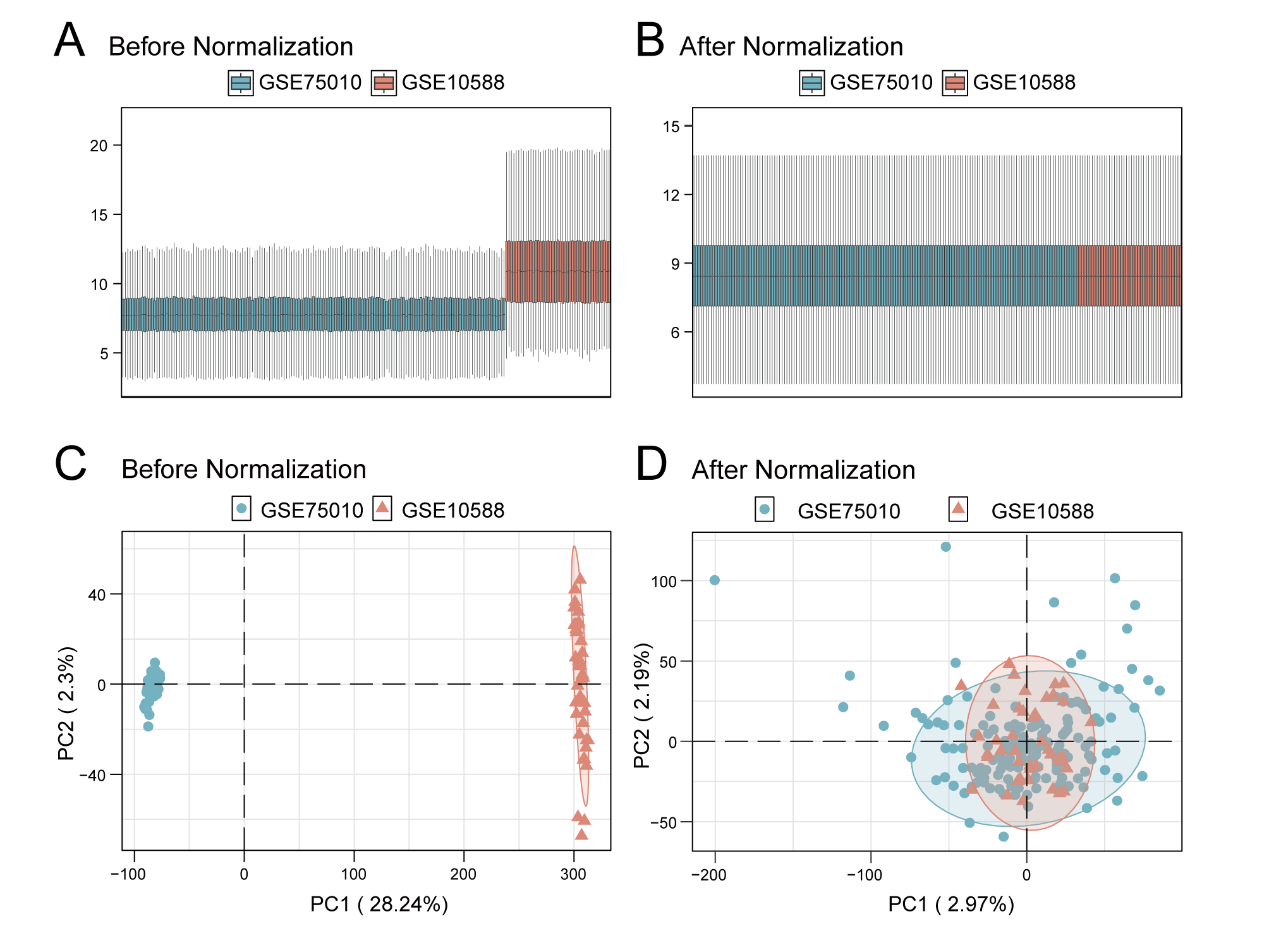
**

**Figure S1. Batch effect correction in the integrated PE dataset.** A. Expression distribution boxplots pre-batch correction. B. Post-correction boxplots demonstrating harmonized distributions. C. PCA plot pre-correction showing batch-driven separation (PC1 vs. PC2). D. Post-correction PCA plot with overlapping sample clusters. The blue represents the dataset GSE75010, and the red represents the dataset GSE10588.

**
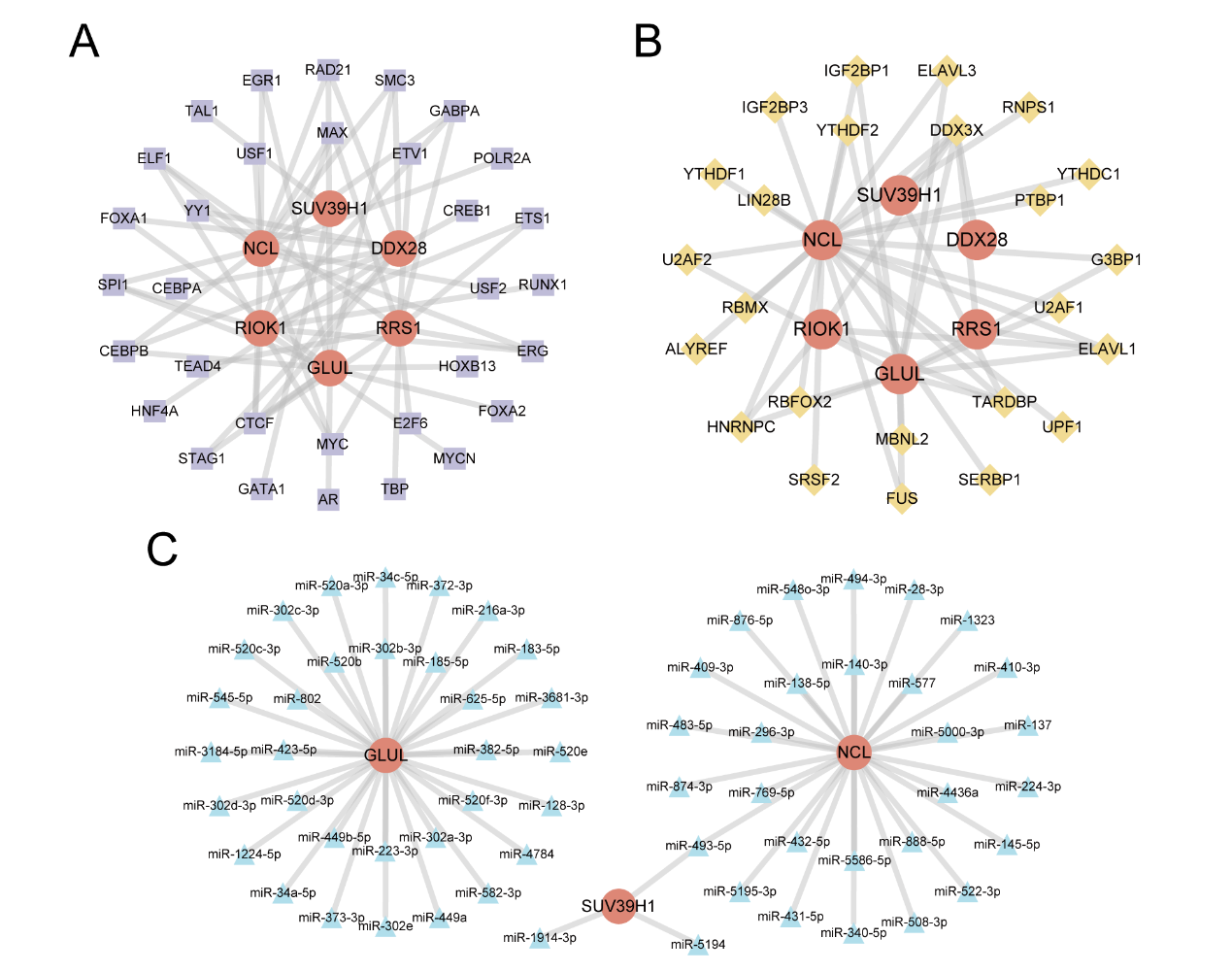
**

**Figure S2. Multi-layered regulatory networks of key genes.**

A. mRNA-TF interaction network: mRNAs (red circles), TFs (purple squares). B. mRNA-RBP network: mRNAs (red circles), RBPs (yellow diamonds). C. mRNA-miRNA network: mRNAs (red circles), miRNAs (blue triangles).
